# The relationship between plasma β-hydroxybutyric acid and conjugated linoleic acid in milk as a biomarker for early diagnosis of ketosis in postpartum Polish Holstein-Friesian cows

**DOI:** 10.1186/s12917-019-2131-2

**Published:** 2019-10-25

**Authors:** Kamila Puppel, Marcin Gołębiewski, Paweł Solarczyk, Grzegorz Grodkowski, Jan Slósarz, Małgorzata Kunowska-Slósarz, Marek Balcerak, Tomasz Przysucha, Aleksandra Kalińska, Beata Kuczyńska

**Affiliations:** Animal Breeding and Production Department, Warsaw University of Life Sciences, Ciszewskiego 8, 02-786 Warsaw, Poland

**Keywords:** Cow, β-Hydroxybutyric acid, Conjugated linoleic acid, Ketosis, Milk

## Abstract

**Background:**

The aim of this study was to investigate the association between plasma β-hydroxybutyric acid (BHBA) and conjugated linoleic acid in postpartum Polish Holstein-Friesian (PHF) cows. The experiment was carried out at an experimental dairy farm, where a herd of approximately 350 cows was kept. Samples were taken at six time points: between days 5–7, 8–14, 15–21, 22–28, 29–35, and 36–42, resulting in 510 samples of both milk and blood. The cows involved in the experiment were divided into two groups – ketotic and healthy – by taking into account general health symptoms, blood serum BHBA, and non-esterified fatty acids (NEFA) concentration at 5–7 days postpartum.

**Results:**

In the first week of lactation, at 5–7 day in milk (DIM), the study showed a 53% lower level of C18:2 *cis*-9 *trans*-11 (CLA9) and an 80% lower level of C18:2 *trans*-10 *cis*-12 (CLA10) in cows with diagnosed ketosis compared to healthy cows. In the second week of lactation (8–14 DIM), a 34% lower level of CLA9 and a 54% lower level of CLA10 was found in the group of cows with BHBA levels > 1.2 mmol/L. Additionally, *Pearson correlation* analysis showed significant negative correlation between BHBA x CLA9 and BHBA x CLA10 in the first week of lactation: − 0.732and − 0.821, respectively.

**Conclusion:**

The study shows that that both CLA9 and CLA10 can be used as markers for the early diagnosis of elevated blood levels of BHBA in postpartum Polish Holstein-Friesian cows.

## Background

Ketosis is considered to be the most important metabolic disease affecting dairy herds, surpassing ruminal acidosis and milk fever [1]. In regards to diagnosis, ketosis can be divided into clinical ketosis, in which the symptoms are easy to recognize and diagnose, and subclinical ketosis, which is more common in high-production farms. In subclinical ketosis, there is an elevated level of ketone bodies and a reduced level of glucose, however, the cow does not yet show signs of clinical ketosis. Subclinical ketosis can occur in up to 60% of cows in a herd, and clinical ketosis from 2 to 15% [2]. Ketone bodies are a group of organic compounds that are intermediate metabolites of fat. These include acetone (formed as a result of spontaneous decarboxylation of acetoacetate), acetoacetic acid (in the form of anion- acetoacetate) and β-hydroxybutyric acid (BHBA, in the form of anion- β-hydroxybutyrate).

The profiles of milk fatty acids are closely related to energy balance in dairy cows, and milk fatty acids are interesting biomarkers for ketosis and negative energy balance (NEB) [3]. The fatty acid synthesis pathway involves the following steps: activation (acetyl-CoA carboxylation), elongation (malonyl-CoA pathway), condensation, reduction, dehydration and another reduction [4–7]. During NEB, NEFA in blood plasma increase and the fatty acid supply to the mammary glands is altered. NEFA released from lipolysis are mainly C16:0, C18:0 and C18:1 *cis*-9, with a further possible conversion of C18:0 to C18:1 *cis*-9 in the mammary glands through the action of Δ9-desaturase [8]. Milk fatty acids are of growing interest in the detection of elevated blood plasma BHBA and NEFA [9–11]. There are four major pathways that create milk fatty acids: diet, de novo synthesis in the mammary glands, and formation in the rumen by biohydrogenation or bacterial degradation [12]. According to Čejna and Chladek [13], the NEB stage is associated with a high ratio of growth hormone to insulin in the blood, which induces the mobilization of long-chain fatty acids from fatty tissue. Puppel et al. [14] reported that C18:1 *cis*-9 may be used as a biomarker for the early diagnosis of elevated blood levels of NEFA during the early stages of lactation in high-yield PHF cows. The highest levels of NEFA in the blood were associated with the highest content of C18:1 *cis*-9 in milk fat, which exceeded 24 g/100 g of fat.

During ketogenesis, mitochondrial β-oxidation of long-chain acids occurs in the liver. According to Foster [15], long-chain fatty acids are transported to mitochondria via carnitine-palmitoyltransferase, which is regulated by the concentration of malonyl-CoA. The first step of ketogenesis relies on the condensation of two molecules of acetyl-CoA to form acetoacetyl-CoA. The third acetyl-CoA molecule is attached to 3-hydroxy-3-methylglytaryl-CoA (HMG-CoA) by mitochondrial HMG-CoA synthetase. Then, HMG-CoA is converted into acetoacetate by HMG-CoA lyase. In turn, acetoacetate is reduced to BHBA by NADH-dependent β-hydroxybutyrate dehydrogenase [16]. The rate of formation of ketone bodies is conditioned by the rate of the conversion of fatty acids into ketone bodies rather than the spawning of the β-oxidation process [17]. In turn, high concentrations of ketone bodies decrease the rate of β-oxidation of fatty acids [18–20].

BHBA is of a higher concentration in the blood of cows that have energy deficiency and is considered to be an indicator of subclinical ketosis [21]. Studies have shown that clinical ketosis in dairy cows generally occurs between week two and seven after calving [22]. BHBA concentrations of < 2.6 and > 1.2 mmol/L in the first week of the postpartum period are indicative of subclinical ketosis [21–23]. BHBA is synthesized both in the process of ketogenesis, as well as in the rumen through butyrate-producing bacteria [23]. Additionally, Melendez et al. [24] reported that all bacteria that produced CLA *cis*-9, *trans*-11 from linoleic acid were butyrate producers.

Ketosis can be monitored using blood, urine, or milk samples. The method of diagnosing ketosis, based on the milk fat/protein ratio, is limited: sensitivity of 58% and specificity of 69% [1]. Periodic herd blood testing for BHBA concentration is the easiest way to early detection of ketosis in cattle, but BHBA in plasma is not a cost effective or convenient analysis [25]. Various cowside tests are available to monitor the ketosis of dairy herds. However, none of the cowside tests have a perfect sensitivity and specificity compared to blood BHBA [26–28]. Therefore, it is important that new tests with greater sensitivity and specificity need to be developed.

### The aim of this study

The aim of this study was to investigate the association betweenplasma BHBA and conjugated linoleic acid in postpartum PHF cows.

## Results

**Fig. 1:**
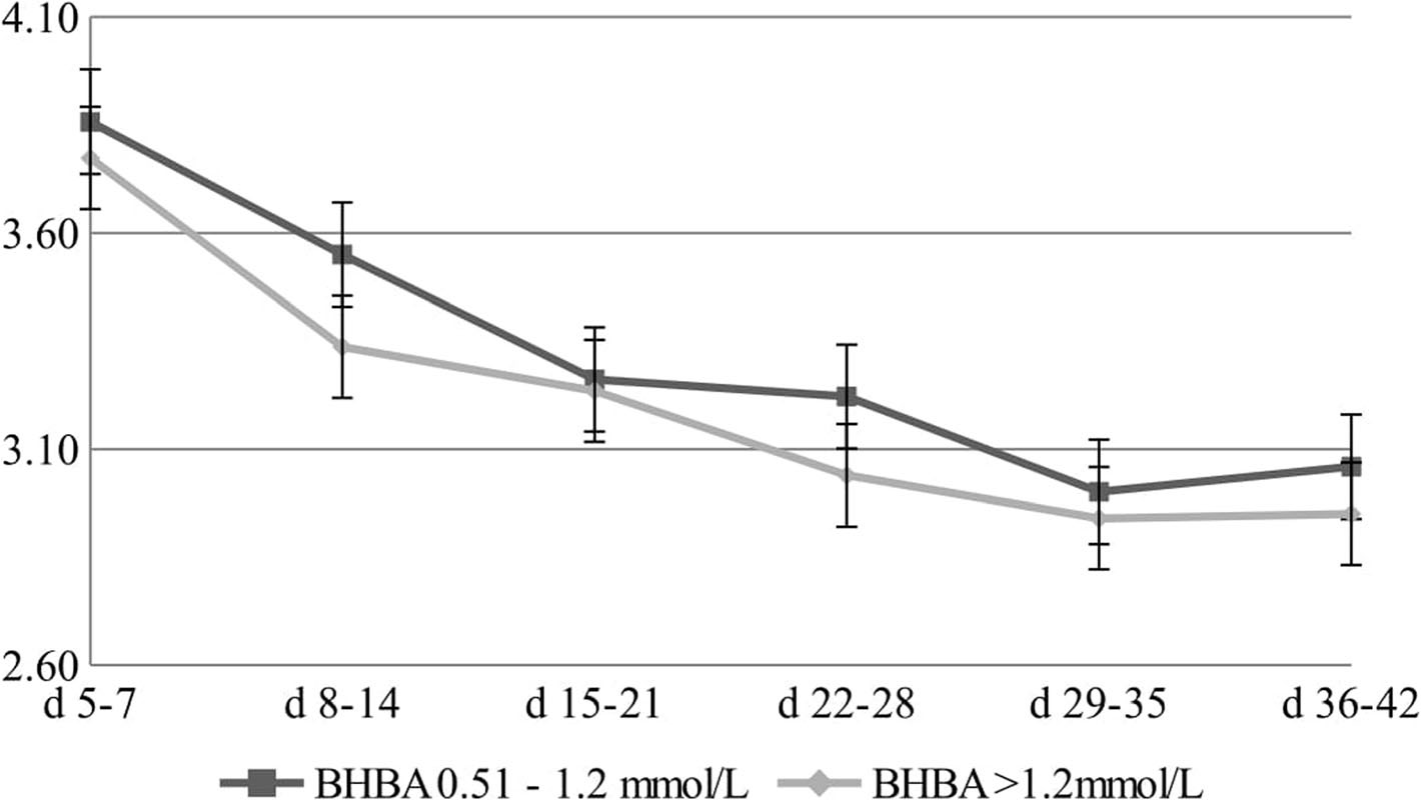
Changes in protein level [%] for different concentrations of BHBA

**Fig. 2:**
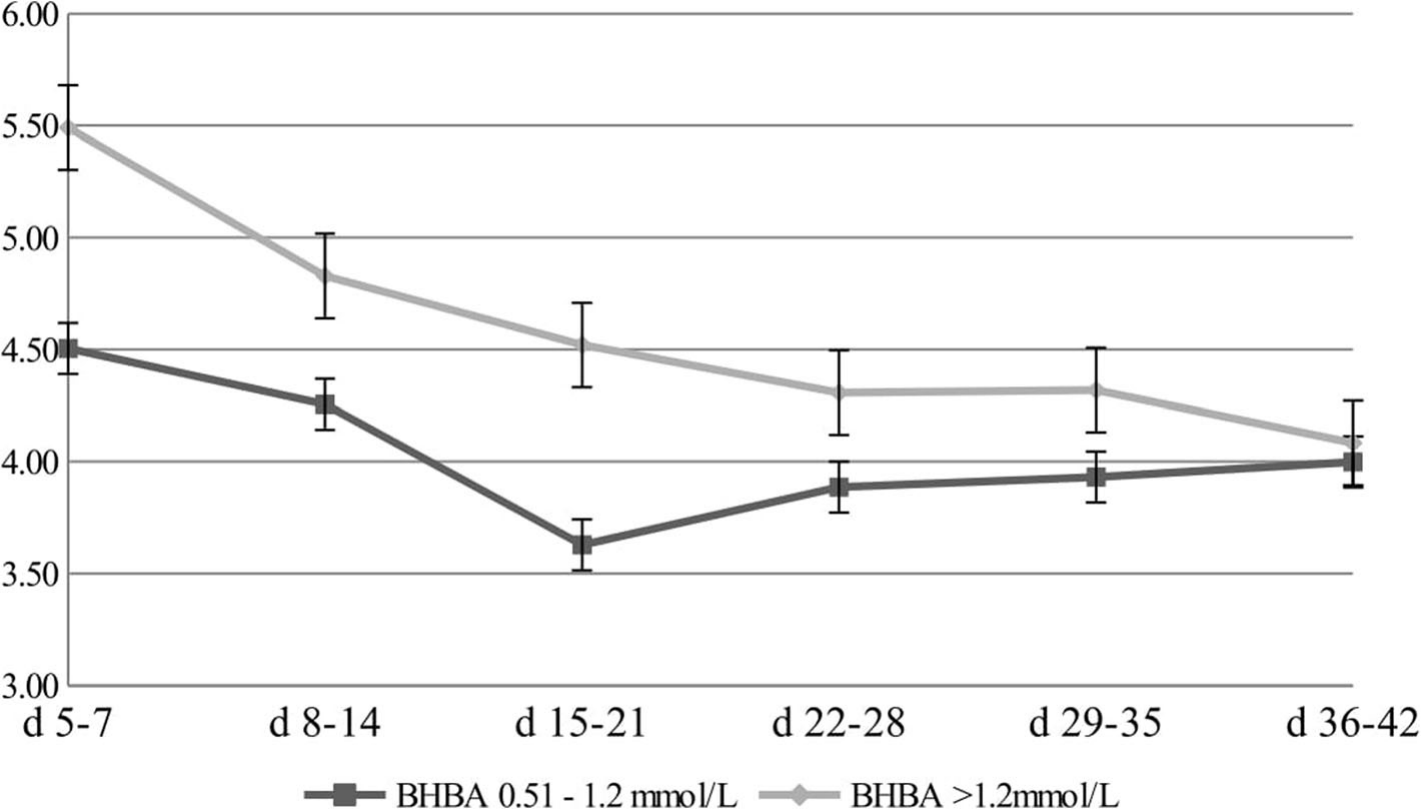
Changes in fat level [%] for different concentrations of BHBA

**Fig. 3:**
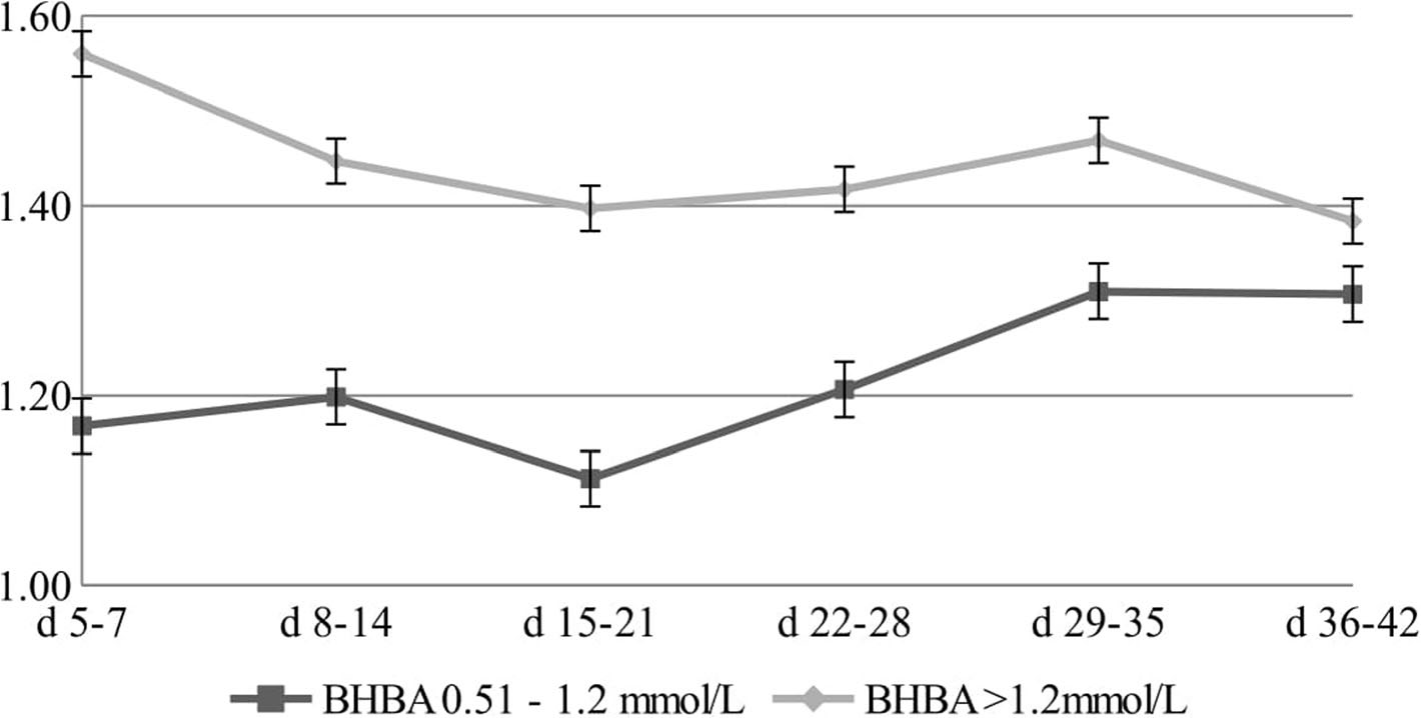
Changes in F/P ratio for different concentrations of BHBA

Figures 1, 2 and 3 show changes in gross milk composition in the first 42 days of lactation. The protein content of the milk at 5–7 DIM was 3.77% in cows with a BHBA level > 1.2 mmol/L, and gradually decreased to a level of 2.94% at 36–42 DIM (Fig. 1). The fat content of the milk at 5–7 DIM was 5.49% in cows with a BHBA level > 1.2 mmol/L. Over the next few weeks, the fat concentration decreased to a level of 4.08% at 36–42 DIM (Fig. 2). The highest F/P ratio (1.56) was demonstrated in the first period – between the fifth and seventh days of lactation in cows with a BHBA level > 1.2 mmol/L (Fig. 3).

In the Table 1 concentration of selected fatty acid (g/100gof fat) of both group in the first 14 days of lactation has been presented. Studies have shown that the concentrations of C4:0, C6:0, C8:0, C10:0, C12:0 and C14:0 are significantly influenced by BHBA.

**Table 1:** Changes in selected fatty acid composition [g/100 g of fat] for different concentrations of BHBA in the first 14 days of lactation

Component	BHBA 0.51–1.2 mmol/L	BHBA > 1.2 mmol/L	BHBA 0.51–1.2 mmol/L	BHBA > 1.2 mmol/L	SEM
	d 5–7		d 8–14		
C4:0	2.821^A^	2.392^A^	2.920 ^A^	2.475 ^A^	0.0963
C6:0	1.550 ^A^	1.356 ^A^	1.761 ^A^	1.296 ^A^	0.0960
C8:0	1.239 ^A^	0.902 ^A^	1.269 ^A^	1.028 ^A^	0.0768
C10:0	2.379 ^A^	1.830 ^A^	1.767 ^A^	2.014 ^A^	0.1154
C12:0	2.718^a^	2.528^a^	2.653^a^	2.468^a^	0.1311
C14:0	9.357^a^	8.818^a^	9.239	9.094	0.3331
C16:0	29.801	30.440	32.420	31.001	0.8070
C16:1	1.495^A^	2.004^A^	1.858	1.840	0.0131
C17:0	0.624	0.590	0.627	0.619	0.0240
C18:0	17.734	17.908	17.601	17.834	0.2729
C18:1 c9	23.581^A^	25.241^A^	25.360^A^	27.262^A^	0.6752
C18:3 n6	0.032^A^	0.049^A^	0.054	0.050	0.0063
C20:4 n6	0.166^A^	0.137^A^	0.161	0.158	0.0072
C20:5 n3	0.088	0.076	0.090	0.087	0.0040
C22:5 n3	0.069	0.065	0.055	0.045	0.0008

Samples of milk and blood were collected from the cows for laboratory analyses at weekly intervals. Samples were taken at six time points: between days 5–7, 8–14, 15–21, 22–28, 29–35, and 36–42. The cows involved in the experiment were divided into two groups – ketotic (BHBA > 1.2 mmol/L and NEFA ≥0.7 mmol/L at 5–7 days postpartum) and healthy (BHBA 0.51–1.2 mmol/L and NEFA < 0.7 mmol/L at 5–7 days postpartum)

Data were presented as LSM with SEM

*NEFA* Nonesterified fatty acids, *BHBA* β-hydroxybutyric acid, *DIM* Days in milk, *LSM* Least square of mean, *SEM* Standard error of LSM

^aA^Means in the same rows (for selected DIM) marked with the same letters differ significantly at: small letters –*P* ≤ 0.05; capitals– *P* ≤ 0.01

**Fig. 4:**
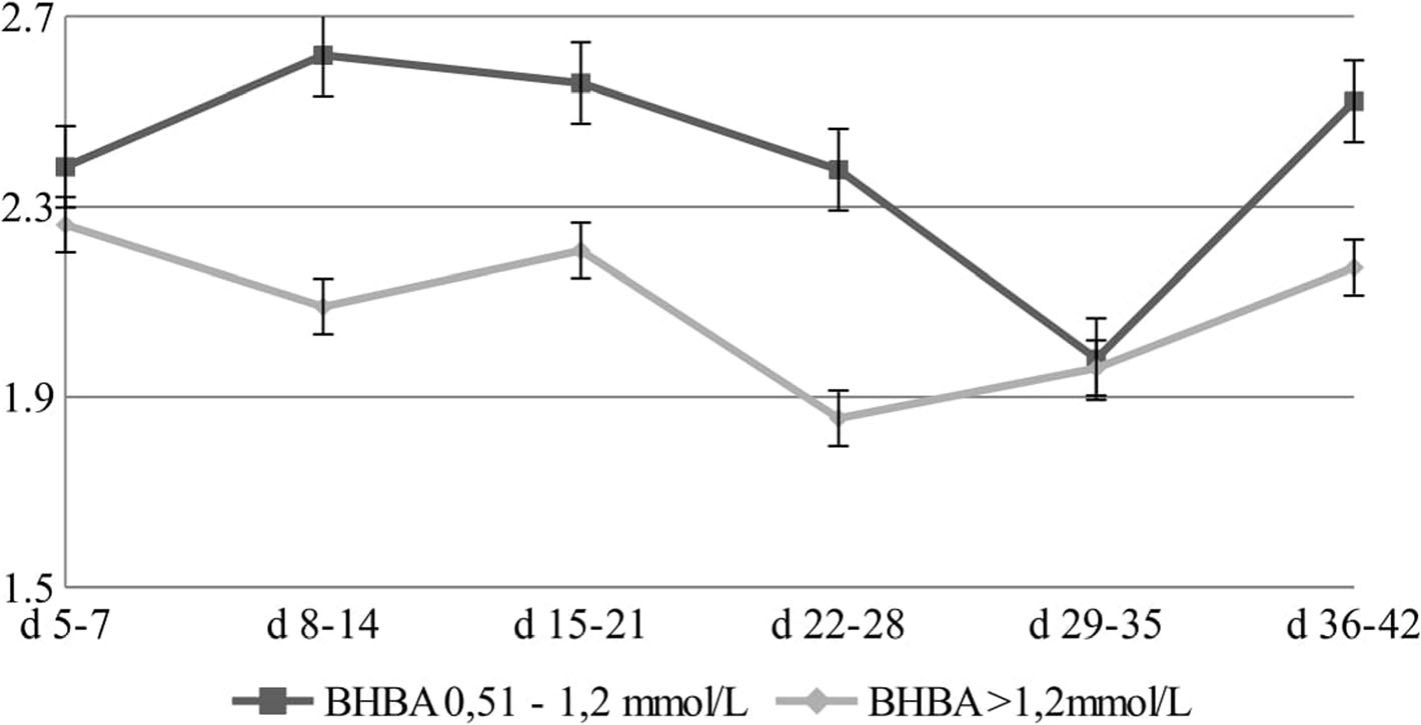
Changes in the concentration of LA [g/100 g of fat] for different concentrations of BHBA

For the first week of lactation (5–7 DIM), studies have shown a 5% lower level of C18:2 n-6 (LA) in cows with a BHBA level > 1.2 mmol/L. In the second week of lactation (8–14 DIM), a 20% higher level has been found in cows with a BHBA concentration in the range of 0.51–1.2 mmol/L (Fig. 4). LA was significantly influenced by both, the lactation phase and BHBA.

**Fig. 5:**
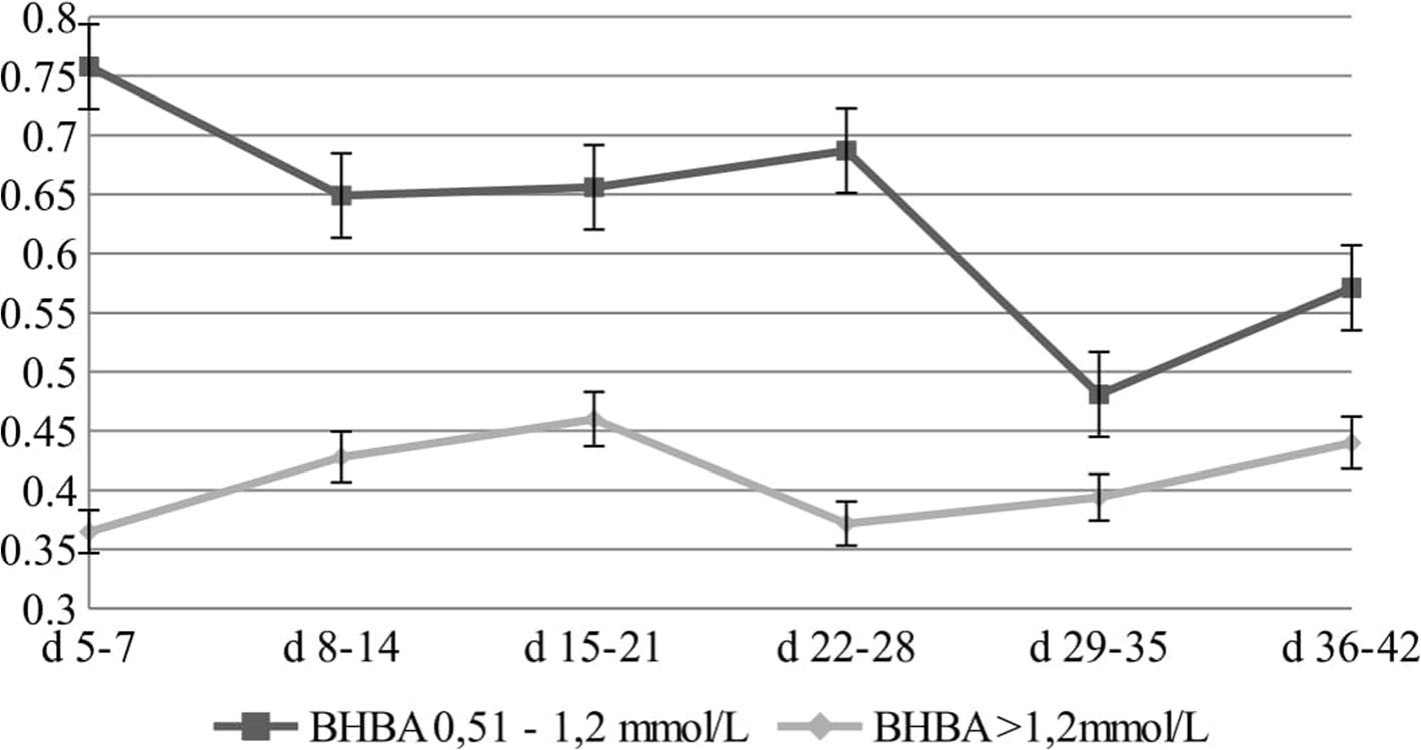
Changes in the concentration of CLA9 [g/100 g of fat] for different concentrations of BHBA

The first week of lactation (5–7 DIM) showed a 51% lower level of C18:2 *cis*-9 *trans*-11(CLA9) in cows with a BHBA level > 1.2 mmol/L– so in cows with diagnosed ketosis. However, in the second week of lactation (8–14 DIM), a 34% lower level in the second group was demonstrated (Fig. 5). There were significant differences in the CLA9 content between the analysed groups.

**Fig. 6:**
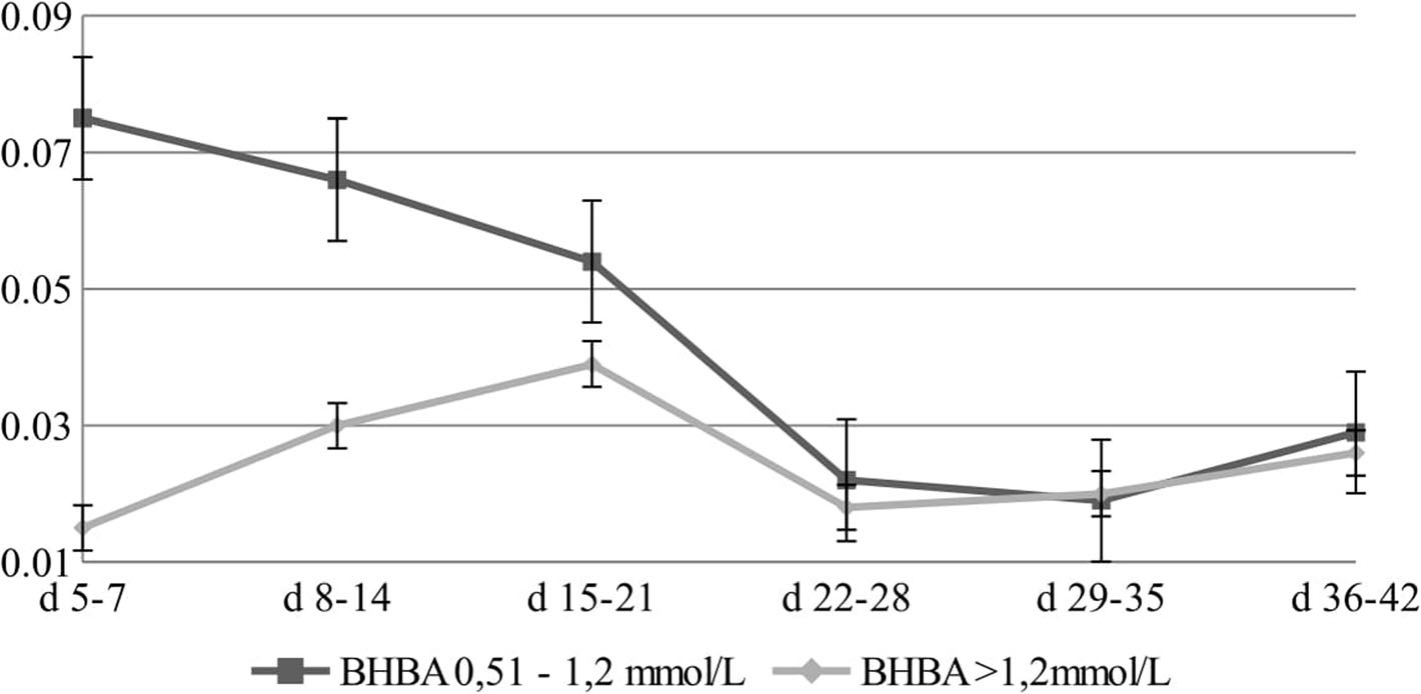
Changes in the concentration of CLA10 [g/100 g of fat] for different concentrations of BHBA

The first week of lactation (5–7 DIM) showed an 80% lower level of C18:2 *trans*-10 *cis*-12 (CLA10) in cows with a BHBA level > 1.2 mmol/L. In the second week of lactation, a 54% lower level was also found in this group (Fig. 6). There were significant differences in the content CLA10 between the analysed groups.

Pearson correlation analysis showed a significant negative correlation between BHBA levels and the levels of selected functional fatty acids (Tables 2 and 3). Strong negative correlation in the first week of lactation (5–7 DIM) was found between BHBA x CLA9 (− 0.732; *p* ≤ 0.01) and BHBA x CLA10 (− 0.821; p ≤ 0.01), and in the second week of lactation between BHBA x CLA9 (− 0.520; p ≤ 0.01) and BHBA x CLA10 (− 0.635; p ≤ 0.01).

**Table 2:** Pearson correlations between individual components during the first week of lactation

	Casein	Protein	Fat	LA	CLA9	CLA10	BHBA	NEFA
Casein	1	0.953^a^	0.054	0.219	−0.039	−0.269^b^	−0.527^a^	−0.254
Protein	0.953^a^	1	−0.113	0.404^a^	0.021	− 0.240	− 0.577^a^	− 0.435
Fat	0.054	− 0.113	1	− 0.628^a^	− 0.311^b^	− 0.165	0.591^b^	0.622^a^
LA	0.219	0.404^a^	−0.628^a^	1	0.664^a^	0.213	−0.418^a^	−0.753
CLA9	−0.039	0.021	−0.311^b^	0.364^a^	1	0.831^a^	−0.732^a^	−0.312
CLA10	−0.269^b^	−0.240	− 0.165	0.213	0.831^a^	1	−0.821^a^	0.044
BHBA	−0.527^a^	−0.577^a^	0.591^b^	−0.418^a^	− 0.732^a^	−0.821^b^	1	0.613^a^
NEFA	−0.254	−0.435	0.622^a^	−0.753	− 0.312	0.044	0.613^a^	1

*LA* C18:2 n-6, *CLA9* C 18:2 *cis*-9 *trans*-11, *CLA10* C18:2 *trans*-10 *cis*-12, *BHBA* β-hydroxybutyric acid, *NEFA* Nonesterified fatty acids

^a^ Correlation significant at a 0.01 level (two-sided)

^b^ Correlation significant at a 0.05 level (two-sided)

**Table 3:** Pearson correlations between individual components during the second week of lactation

	Casein	Protein	Fat	LA	CLA9	CLA10	BHBA	NEFA
Casein	1	0.923^a^	− 0.066	0.228^b^	0.251^b^	0.100	−0.292^a^	−0.359
Protein	0.923^a^	1	−0.328^a^	0.265^b^	0.086	−0.035	−0.373^a^	− 0.270^a^
Fat	−0.066	− 0.328^a^	1	− 0.015	0.147	0.217	0.295^a^	0.490^a^
LA	0.228^b^	0.265^b^	−0.015	1	0.532^a^	0.401^a^	−0.553^a^	−0.630^a^
CLA9	0.251^b^	0.086	0.147	0.532^a^	1	0.848^a^	−0.520^a^	−0.206
CLA10	0.100	−0.035	0.217	0.401^a^	0.848^a^	1	−0.635^a^	−0.060
BHBA	−0.292^a^	−0.373^a^	0.295^a^	−0.553^a^	− 0.520^a^	−0.635^a^	1	0.632^a^
NEFA	−0.359	−0.270^a^	0.490^a^	−0.630^a^	− 0.206	−0.060	0.632^a^	1

*LA* C18:2 n-6, *CLA9* C 18:2 *cis*-9 *trans*-11, *CLA10* C 18:2 *trans*-10 *cis*-12, *BHBA* β-hydroxybutyric acid, *NEFA* Nonesterified fatty acids

^a^ Correlation significant at a 0.01 level (two-sided)

^b^ Correlation significant at a 0.05 level (two-sided)

## Discussion

Clinical ketosis most frequently occurs in susceptible high-yield dairy cows in the first days of lactation as a consequence of inadequate nutrition and management [9, 19, 21]. Clinical features include anorexia, depression, and metabolic disease. Cows also have low milk production and poor reproductive capacity [29]. High levels of BHBA induce hepatic oxidative stress, apoptosis, and inflammation [30]. Therefore, rapid diagnosis of ketosis is very important.

There were significant differences in the protein content between the analysed groups. Similar relations were demonstrated by Ikoen et al. [31] and Peckaet al [32].. These authors found that the protein concentration in milk stabilizes after the sixth week of lactation. Based on the results obtained, it can be concluded that the reduction in the level of protein in subsequent collections was caused by a dilutioneffect, and the reduction in the concentration of this milk ingredient was the result of an increase in the cows’ total milk yield.

Fourier transform infrared spectrum of milk and milk composition could be used by breeders to predict blood BHBA levels, because these data are available during Dairy Herd Improvement testing [33]. The protein content of the milk at 5–7 DIM was 3.77% in cows with a BHBA level > 1.2 mmol/L, and gradually decreased to a level of 2.94% at 36–42 DIM (Fig. 1). Heuer et al. [34] suggested that changes in gross composition of milk are useful risk predictors for energy balance in early lactation, e.g. fat/protein ratio > 1.4, milk protein < 2.9%, and milk fat > 4.8%.

There were significant differences in the fat content of milk between the analysed groups. The fat content of the milk at 5–7 DIM was 5.49% in cows with a BHBA level > 1.2 mmol/L. Duffield et al. [35] showed a direct relationship between BHBA and fat content. Cows with a BHBA level > 0.7 mmol/L were characterized by a fat concentration > 3.60%. Additionally, after analysing the results, it was also confirmed that the milk fat showed the least stability among all milk components. Other authors have come to similar conclusions [36–38].

The optimum fat/protein (F/P) ratio is 1.2–1.4. Lower values are the result of subclinical rumen acidosis, which can endanger the reproduction performance of cows and enhance the possible development of mineral metabolism disorders. On the other hand, an F/P ratio higher than 1.4 is connected with deficiency of energy and subclinical ketosis [13]. The highest F/P ratio (1.56) was demonstrated in the first period – between the fifth and seventh days of lactation in cows with a BHBA level > 1.2 mmol/L (Fig. 3). García et al. [39] reported a positive relationship between BHBA levels and the F/P ratio (*R*2 = 0.42). Herds with ketosis problems in early lactation tend to have an increased probability of occurrence of displaced abomasum and herd removal (> 8%) in the initial phase of lactation [1]. Additionally, Toni et al. [40] concluded, that cows with an F/*P* > 2.0 during early lactation showed an increase in postpartum diseases such as retained placenta, metritis, and clinical endometritis.

NEB is partly caused by cows limiting their intake of dry matter during the postpartum period. The lactation phase, as well as NEB stage, significantly contribute to changes in the composition of milk fat, as well as limit the activity of individual fatty acid pathways. About 50% of the fatty acids in milk originate from the diet, while the remaining 50% is from adipose tissue, but the adipose tissue contribution is much higher during early lactation [41]. Dairy cow in NEB is predisposed to hepatic lipidosis and ketosis, because of the inability to dispose of mobilized FA via β-oxidation or the limited capacity to export FA reesterified into TAG from the liver [42]. Studies have shown that the concentrations of C4:0, C6:0, C8:0, C10:0, C12:0 and C14:0 are significantly influenced by BHBA. Substrates for de novo synthesis of FA are acetate and β-hydroxybutyrate, used by the mammary epithelial cells to synthesize short- and medium-chain fatty acids and part of the 16-carbon FA [43]. Van Knegsel et al. [44] suggested that during the NEB period, the de novo synthesis of FA is reduced and the cow’s body begins to use its own reserves, which is confirmed by the obtained results.

Studies have shown that the concentration of C18:2 n-6 was significantly influenced by both the lactation phase and BHBA. C18:2 n-6 acts as a substrate for C18:1 *trans*-11, which is transformed into C18:2 *cis*-9 *trans*-11, as well as for long-chain fatty acids formed by desaturation and elongation [45–47]. Gross et al. [3] showed that a lowered concentration of unsaturated fatty acids was associated with the stabilization of the cows’ energy balance.

Isomers C18:2 *cis*-9 *trans*-11 are formed in ruminant tissue and the mammary glands via the action of stearoyl-CoA desaturase on C18:1 *trans*-7 and C18:1 *trans*-11, respectively [46, 47]. Lock and Garnsworthy [48] estimated that, the endogenous synthesis of CLA to be > 80% of the total concentration. Rumen pH has a significant role in keeping a viable rumen environment appropriate for *B. fibrisolvens* involved in the biohydrogenation of C18:2 n-6 and C18:3 n-3. Additionally, ruminal pH at 6.0 or above has a positive effect on C18:1 *trans*-11 and C18:2 *cis*-9 *trans*-11 contents in rumen cultures [49]. On the other hand, decreased in rumen pH results in bacterial population changes and consequent modification end products of fermentation [50]. When ketosis occurs, high levels of ketone bodies inhibit the activity of acetyl-CoA and so decrease the transport of acetyl-CoA to ketone bodies, which may result in acetyl-CoA accumulating quickly. In this study, the level of C18:2 *cis*-9 *trans*-11 was significantly influenced by the concentration of BHBA in the blood plasma. Melendez et al. [24] also reported that early postpartum cows with a plasma BHBA level > 0.7 mmol/L tended to have a lower proportion of CLA than cows with a BHBA level ≤ 0.7 mmol/L. Dietary supplementation of plant and animals oils or pasture results in substantial increases in the concentration of CLA in milk fat [51–53], as does microbial activity in the rumen [47]. In our case, there was nothing in the diet composition to suggest a bypass form, nor was there an excessive amount of dietary fat that would be available to microbes in the rumen to generate CLA. Therefore, the decline of CLA concentrations in milk is likely due to a lower supply from the rumen.

Concentrations of NEFAs and BHBA are the basic elements of the metabolic profile, which are used in the diagnostics of metabolic diseases [50]. However, Duffield [54], reported that the use of NEFA is a better indicator of energy imbalance in prepartum animals than BHBA, but BHBA is more useful postpartum. In the present study, a significant positive correlation was established between NEFA and BHBA (Tables 2 and 3). Duffield [54] demonstrated, that a 1% increase in milk fat was associated with 2-fold increase in the risk of subclinical ketosis. Additionally, changes in rumen biohydrogenation increase the molar proportions of trans fatty acids that inhibit milk fat synthesis [55, 56]. The increased in the concentration of BHBA and NEFA due to the mobilization of endogenous lipids, leads to a decrease in the percentage of milk protein [57, 58]. Pearson correlation analysis showed a significant negative correlation between BHBA levels and the levels of selected functional fatty acids (Tables 2 and 3).

## Conclusion

Despite the advantages or disadvantages of urine or milk tests, none of them have perfect sensitivity and specificity compared to the “gold standard” test of blood BHBA. Therefore, it is important that new tests with greater sensitivity and specificity need to be developed. As shown in the study results, the high concentration of BHBA in postpartum cows was associated with significantly lower levels of LA, CLA9 and CLA10.Strong negative correlation in the first week of lactation was found between BHBA x CLA9 (− 0.732) and BHBA x CLA10 (− 0.821), and in the second week of lactation between BHBA x CLA9 (− 0.520) and BHBA x CLA10 (− 0.635). Additionally, analytical devices equipped with Fourier Transform Spectrometer (FTIR) allow to determine parameters such as: fat, protein, lactose and fatty acids: CLA9, MUFA, PUFA, SFA in a very short time. Fourier transform infrared spectrum of milk composition could be used by breeders to predict blood BHBA levels, because these data are available during Dairy Herd Improvement testing. Limiting the frequency and amount of blood sampling for laboratory analysis, and replacing them with standard milk analysis using the FTIR technique will improve welfare by reducing the number of stress factors, as well as influence faster diagnostics of metabolic disorders. The demonstrated negative relationship between plasma BHBA levels in blood and CLA levels in milk remains another reason to consider the fundamental prevention of high ketone levels when the dairy industry is looking for milk and its derivatives with high C18:2 *cis*-9 *trans*-11 and C18:2 *trans*-10 *cis*-12 levels.

Due to the obtained high level of correlation coefficient, repeatability and representative number of samples, it can be stated that C18:2 *cis*-9 *trans*-11 and C18:2 *trans*-10 *cis*-12 are useful markers for the early diagnosis of elevated blood levels of BHBA in postpartum Polish Holstein-Friesian cows.

## Methods

### Study design

The experimental procedures were carried out according to the regulations of the Polish Council on Animal Care and were approved by the Warsaw University of Life Sciences Care Committee. The experiment was carried out at the experimental dairy farm of the Warsaw University of Life Sciences (WULS), in which a herd of approximately 350 cows was kept in free stall housing system, with an average performance exceeding 10,000 kg of milk per lactation. Table 4 shows the lactating cows’ nutrient requirement.

**Table 4:** Lactating cows’ nutrient requirement

Cow description	
Cow weight	680 kg
Lactation days	50
Milk production	38 kg
Milk fat	4.00%
Milk protein	3.07%
Nutrient requirement	
NEL (Mcal/day)	39.5
Metabolic protein (g/day)	2589
Ca (g/day)	65
F (g/day)	59
K (g/day)	228

*NEL* Netto energy lactation, *Ca* Calcium, *F* Phosphorus, *K* Potassium

The cows’ feeding regime was based on the total mixed ration (TMR) diet (ad libitum) (Table 5). Cows were fed twice a day. Dry matter intake was determined weekly by weighing remaining orts.

**Table 5:** Ingredient and chemical composition of TMR

	TMR diet
Ingredient [kg/d DM]	
Maize silage	9.12
Alfalfa silage	3.50
Corn silage	2.35
Soybean meal	2.62
Pasture ground chalk	0.20
VIT-RA BML- vitamin mix	0.20
Salt	0.05
Rapeseed meal	2.07
Magnesium oxide	0.07
Chemical composition [g/kg DM]	
Ash	5.25
Crude protein	15.85
Fat	4.89
Starch	289.21
Sugar	77.45
ADF	30.02
NDF	41.21
Ca	0.90
P	0.61
NEL (Mcal/kg)	1.67
Total, kg of DM (offered)	20.18
Daily intake (kg)	19.95
Average milk production (kg)	34.18
UFL balance (%)	3.25
PDIN	2.58
PDIE	−2.22

VIT-RA BML (values per kg): 150 g Ca, 100 g P, 50 g Na, 40 g Mg, 9000 mg Zn, 7000 mg Mn, 1000 mg Cu, 100 mg J, 50 mg Se, 1,200,000 IU vitamin A, 120000 IU vitamin D_3_, 5000 mg vitamin E, 93 mg vitamin K, 80 mg vitamin B_1_, 160 mg vitamin B_6_, 110 mg vitamin B_2_, 1000 μg vitamin B_12_ (PPH VITRA, Kusowo, Poland)

*TMR* Total mixed ration, *DM* Dry matter, *ADF* Acid detergent fiber, *NDF* Neutral detergent fiber, *NEL* Netto energy lactation, *Ca* Calcium, *F* Phosphorus, *K* Potassium, *UFL* Unit of milk production, *PDIN* Protein digested in the small intestine when rumen-fermentable nitrogen is limiting, *PDIE* Protein digested in the small intestine when rumen-fermentable energy is limiting

During the health monitoring procedure of all herd (360 cows), 85 cows were used in this study. These cows were multiparous, in the second lactation, and had an average body weight of 682.45 kg. Based on the clinical symptoms (reduced feed intake and milk yield) and serum BHBA and NEFA concentration, this group of cows included 40 ketotic cows whose serum BHBA concentration was > 1.2 mmol/L and NEFA ≥0.7 mmol/L at 5–7 days postpartum, and 45 healthy cows whose serum BHBA concentration was 0.51–1.2 mmol/L and NEFA < 0.7 mmol/L at 5–7 days postpartum. The characteristics of the ketotic and healthy cows are presented in Table 6.

**Table 6:** Characteristics of healthy and ketotic cows

Item	Ketosis (*n* = 40)		Healthy (*n* = 45)		*P*-value
	LSM	SEM	LSM	SEM	
BW	648 kg	0.052	694 kg	0.053	0.012
Milk yield	33.8 kg/day	0.118	34.1 kg/day	0.123	0.456
DMI	18.45 kg/day	0.173	21.86 kg/day	0.119	0.036
BCS	2.32	0.108	3.22	0.065	0.025
NEFA	1.22 mmol/L	0.082	0.35 mmol/L	0.114	< 0.001
BHBA	1.43 mmol/L	0.063	0.68 mmol/L	0.088	< 0.001

*BW* Body weight, *DMI* Dry matter intake, *BCS* Body condition score, *NEFA* Non-esterified fatty acids, *BHBA* β-hydroxybutyric acid, *LSM* Least square of mean, *SEM* Standard error of LSM

Samples of milk and blood were collected from the cows for laboratory analyses at weekly intervals. Samples were taken at six time points: between days 5–7, 8–14, 15–21, 22–28, 29–35, and 36–42, resulting in 510 samples of both milk and blood.

Body condition score was assessed once a week by BCS-5 method describing by Edmonson et al. [59] and Wildman et al. [60].

The cows were milked daily at 05:30 and 17:30, and the milk yield was recorded at each milking. During the experiment, milk samples were obtained from each cow from the morning and evening milking. The samples were combined, giving a representative sample. The milk was placed in sterile bottles, preserved with Mlekostat CC and immediately transported to the Cattle Breeding Division (Milk Testing Laboratory of WULS) for compositional analysis.

Blood samples (10 mL) were taken from each cow by jugular vein puncture using tubes (Vacuette, Germany) containing potassium-EDTA (K3EDTA, 1.8 g/L of blood) as an anticoagulant. Blood samples were centrifuged at 1800×g at 4 °C for 15 min, and the supernatant was immediately transported to the Veterinary Centre of WULS for the analysis of blood plasma metabolites (BHBA and NEFA).

### Chemical analysis

The basic parameters of milk: fat, protein, and casein contents, were determined by automated infrared analysis using a Milkoscan FT 120 analyser (Foss Electric, Hillerød, Denmark).

The level of BHBA and NEFA was determined using a Biochemical analyser BS800M (PZ Cormay, Warsaw, Poland).

Fatty acid methylation was performed according to the *trans-*esterification method EN ISO 5509 [61]. Individual fatty acids were identified in crude fat using an Agilent 7890A GC (Agilent, Waldbronn, Germany) according to Puppel et al. [53]. Each peak was identified using pure methyl ester standards: FAME Mix RM–6, Lot LB 68242; Supelco 37 Comp. FAME Mix, Lot LB 68887; Methyl linoleate, Lot 094 K1497; CLA Conjugated (9Z, 11E), Lot BCBV3726 (Supelco, Bellefonte, PA, USA).

### Statistical analysis

The data obtained were statistically analysed using the IBM SPSS 23.0 package [62]. The distribution of the milk chemical composition and selected fatty acids were checked using the Shapiro-Wilk test. ANOVA analysis was used to establish the influence of the lactation phase on milk chemical composition and the level of selected fatty acids. The changes in concentration of selected fatty acids in regard to BHBA blood level and lactation stage were established by multivariate analysis.

The following statistical model was used:

$$ \mathrm{Y}=\upmu +{\mathrm{A}}_{\mathrm{i}}+{\mathrm{B}}_{\mathrm{j}}+{\left(\mathrm{AxB}\right)}_{\mathrm{i}\mathrm{j}}+{\mathrm{e}}_{\mathrm{i}\mathrm{j}\mathrm{k}} $$

where μ – mean, A_i_ – day in lactation, B_j_– BHBA concentration, AxB – interaction between day in lactation and BHBA concentration, e_ij_– random error. Only the interactions between factors whose influence was statistically significant (*P* ≤ 0.01 or *P* ≤ 0.05) were considered. The level of significance was determined after performing preliminary statistical analyses.

Pearson correlation quantifies the degree of linear relationship between two variables *x* and *y*, and has been used to describe correlation between: casein, protein, fat, LA, CLA9, CLA10, BHB and NEFA.

## Data Availability

All data generated or analyzed during the study are included in this published article. The datasets used and/or analyzed in the current study are available from the corresponding author on reasonable request.

## Abbreviations

BHBA: β-hydroxybutyric acid
CLA10: C18:2 *trans*-10 *cis*-12
CLA9: C18:2 *cis*-9 *trans*-11
LA: C18:2 n-6
NEFA: Nonesterified fatty acids
